# Thioredoxin A regulates protein synthesis to maintain carbon and nitrogen partitioning in cyanobacteria

**DOI:** 10.1093/plphys/kiae101

**Published:** 2024-02-22

**Authors:** Manuel J Mallén-Ponce, Francisco Javier Florencio, María José Huertas

**Affiliations:** Departamento de Bioquímica Vegetal y Biología Molecular, Universidad de Sevilla, 41012 Sevilla, Spain; Instituto de Bioquímica Vegetal y Fotosíntesis (Universidad de Sevilla, Consejo Superior de Investigaciones Científicas), 41092 Sevilla, Spain; Departamento de Bioquímica Vegetal y Biología Molecular, Universidad de Sevilla, 41012 Sevilla, Spain; Instituto de Bioquímica Vegetal y Fotosíntesis (Universidad de Sevilla, Consejo Superior de Investigaciones Científicas), 41092 Sevilla, Spain; Departamento de Bioquímica Vegetal y Biología Molecular, Universidad de Sevilla, 41012 Sevilla, Spain; Instituto de Bioquímica Vegetal y Fotosíntesis (Universidad de Sevilla, Consejo Superior de Investigaciones Científicas), 41092 Sevilla, Spain

## Abstract

Thioredoxins play an essential role in regulating enzyme activity in response to environmental changes, especially in photosynthetic organisms. They are crucial for metabolic regulation in cyanobacteria, but the key redox-regulated central processes remain to be determined. Physiological, metabolic, and transcriptomic characterization of a conditional mutant of the essential *Synechocystis* sp. PCC 6803 thioredoxin *trxA* gene (STXA2) revealed that decreased TrxA levels alter cell morphology and induce a dormant-like state. Furthermore, TrxA depletion in the STXA2 strain inhibited protein synthesis and led to changes in amino acid pools and nitrogen/carbon reserve polymers, accompanied by oxidation of the elongation factor-Tu. Transcriptomic analysis of TrxA depletion in STXA2 revealed a robust transcriptional response. Downregulated genes formed a large cluster directly related to photosynthesis, ATP synthesis, and CO_2_ fixation. In contrast, upregulated genes were grouped into different clusters related to respiratory electron transport, carotenoid biosynthesis, amino acid metabolism, and protein degradation, among others. These findings highlight the complex regulatory mechanisms that govern cyanobacterial metabolism, where TrxA acts as a critical regulator that orchestrates the transition from anabolic to maintenance metabolism and regulates carbon and nitrogen balance.

## Teaser

Thioredoxin A depletion in cyanobacteria leads to protein synthesis inhibition, reserve polymer accumulation, and induction of a robust transcriptomic response that triggers a dormant-like state.

## Introduction

Photosynthetic cell metabolism involves multiple levels of redox reactions that provide energy for the cells while regulating the flux of metabolic pathways. Many of these pathways undergo temporal partitioning into day and night cycles. Redox-active proteins, such as thioredoxins (Trxs), play a crucial role in the determination of metabolic flux by modifying target proteins through thiol modifications. Trxs are small oxidoreductases that contain a redox-active disulfide bond in a conserved WC(G/P)PC motif (Holmgren 1985). Trxs play important functional roles in prokaryotic and eukaryotic organisms, including the reduction and oxidation of cysteine residues and dithiol/disulfide exchange reactions with target proteins (Schürmann and Buchanan 2008). Oxygenic photosynthetic organisms possess different Trx isoforms, which are classified into 7 groups (Trx *h*, Trx *o*, Trx *f*, Trx *z*, Trx *m*, Trx *x*, and Trx *y*; Geigenberger et al. 2017). Three of these isoforms originate from cyanobacteria, namely TrxA or *m-*type, TrxB or *x-*type, and TrxQ or *y-*type (Florencio et al. 2006). Cyanobacteria also have 2 additional Trx groups, TrxC and TrxA3 (López-Maury et al. 2018; Mallén-Ponce et al. 2022).

Early in vitro studies provided evidence for the regulatory role of Trxs in the Calvin Benson (CB) cycle, which includes ATP synthesis and the export of reducing equivalents from chloroplasts (Jacquot et al. 1978; Buchanan et al. 1979). These studies also revealed the molecular mechanism for the light-dependent regulation of different chloroplastidic target proteins. Furthermore, early studies in cyanobacteria demonstrated the ability of Trx to reduce CB cycle enzymes (Yee et al. 1981). Later, genetic studies showed that TrxA is essential for growth in both *Synechocystis* sp. PCC 6803 and *Synechococcus* sp. PCC 6301 (Muller and Buchanan 1989; Navarro and Florencio 1996). *Synechocystis* sp. PCC 6803 (hereafter *Synechocystis*) possesses 1 copy of each of the 4 main types of Trxs: TrxA (*m-*type), TrxB (*x*-type), TrxQ (*y*-type), and TrxC (Florencio et al. 2006), and mutant strains lacking TrxB, TrxQ, and TrxC have provided insights into the specific roles of different Trxs (Pérez-Pérez et al. 2009; López-Maury et al. 2018). A conditional knockdown mutant strain for TrxA (STXA2) was generated, where the *trxA* gene is controlled by the arsenite-inducible promoter P_arsB_ in a neutral site and responds to the presence of arsenite in the medium (hereafter referred to as inducer) (López-Maury et al. 2003; Mallén-Ponce et al. 2021). This mutant shows a strong phenotype when TrxA levels decrease below 10% of those of wild type (WT). Under these conditions, the enzymes fructose-1,6/sedoheptulose-1,7-bisphosphatase (FBP/SBPase) and 2-Cys peroxiredoxin (2-Cys Prx) were identified as TrxA target proteins, demonstrating their participation in activation of the CB cycle and oxidative stress response, respectively (Mallén-Ponce et al. 2021). In contrast to other model cyanobacteria, *Anabaena* sp. PCC 7120 contains 2 TrxA isoforms (Trx-*m*1 and Trx-*m*2), with Trx-*m*1 being more abundant than the other isoform (Mihara et al. 2020). Mutant strains lacking Trx-*m*1 (Δ*trxM1*) or Trx-*m*2 (Δ*trxM2*) were observed to grow similarly to WT under normal photosynthetic conditions (Deschoenmaeker et al. 2018). However, only Δ*trxM1* strain showed substantially suppressed growth under nitrogen-fixing conditions (Deschoenmaeker et al. 2019). In plants, the role of different Trxs has also been analyzed by generating knockdown or knockout mutants (Geigenberger et al. 2017). The *m*-types have been found to contribute ∼70% of the total Trx pool in the Arabidopsis (*Arabidopsis thaliana*) chloroplast (Okegawa and Motohashi 2015). Studies with the *trx m124-2* triple mutant showed that Trx-*m*4 did not accumulate, while Trx-*m*1 and *m2* protein levels were 52% and 13% relative to the WT strain, respectively (Okegawa and Motohashi 2015). Analysis of this strain revealed a substantial *in vivo* role of Trxs in the activation of CB cycle enzymes, export of reducing equivalents through malate and PSII, and cyclic electron transport (Okegawa and Motohashi 2015, 2020).

Cyanobacteria are subject to diurnal light/dark cycles, resulting in important metabolic changes (Welkie et al. 2019). During the light period, cyanobacteria perform photosynthesis, CO_2_ fixation, and de novo synthesis of metabolites (Sarkar et al. 2019; Werner et al. 2019). Recent studies have shown that several proteins in *Synechocystis* sp. PCC 6803, *Synechococcus* sp. PCC 7002, and *Anabaena* sp. PCC 7120 are redox regulated by light/dark cycles, suggesting that the Trx system can modulate the activation of these proteins (Ansong et al. 2014; Guo et al. 2014; Mihara et al. 2020). The redox-sensitive proteins identified in these studies include those involved in amino acid biosynthesis, protein synthesis, and the CB cycle, which is known to be redox-regulated in cyanobacteria. Biochemical studies have shown that translational elongation in *Synechocystis* can be inhibited by reactive oxygen species (ROS), which oxidize elongation factor-Tu (EF-Tu) and elongation factor-G (EF-G) (Kojima et al. 2007, 2009; Yutthanasirikul et al. 2016; Jimbo et al. 2018). Recently, specific cysteine oxidation found in EF-Tu has also been reported to inhibit translation in *Arabidopsis* chloroplasts (Toriu et al. 2023). These findings highlight the critical interaction between Trxs and EF-Tu for the optimal functioning of photosynthesis.

Additionally, cyanobacteria store reserve polymers, such as glycogen, during the light phase, and use them during the dark phase (Díaz-Troya et al. 2014). The transition from the light to the dark phase causes an increase in glycolysis and oxidative pentose phosphate (OPP), which provides a carbon source and energy for cell maintenance in the dark phase (Knowles and Plaxton 2003; Shinde et al. 2020). The glucose-6-phosphate dehydrogenase enzyme (G6PDH), which is the first enzyme in the OPP pathway, has been reported to be regulated by its allosteric activator OpcA (Hagen and Meeks 2001). OpcA can be reduced by Trx-*m*1 or Trx-*m*2, resulting in the inactivation of G6PDH in *Anabaena* (Mihara et al. 2018).

Proteomic, biochemical, and physiological studies have shown that TrxA plays a role in the redox regulation of the CB cycle and antioxidant response enzymes in cyanobacteria (Mihara et al. 2018; Mallén-Ponce et al. 2021; Fukui et al. 2022). However, several approaches have suggested that TrxA is also involved in the regulation of central cellular processes, such as gene transcription and protein biosynthesis. Despite our extensive knowledge of target proteins, our understanding of the mechanisms of TrxA-mediated central regulatory processes remains limited. To this goal and to investigate whether TrxA acts as a regulatory hub across transitions from anabolic to maintenance metabolism in cyanobacteria, physiological, metabolic, and transcriptomic changes in the STXA2 strain were studied under conditions of TrxA depletion and recovery. TrxA depletion resulted in the arrest of anabolic metabolism, followed by the degradation of light-harvesting complexes, known as chlorosis, and the accumulation of nitrogen and carbon reserve polymers leading to a dormant-like state. This metabolic shift was accompanied by a global transcriptomic reprogramming, suggesting a fine-tuned and orchestrated response. The STXA2 analysis confirmed the indispensable role of TrxA in protein synthesis and cell growth. The effect of reintroducing the inducer into the STXA2 strain with undetectable TrxA levels was also investigated. Almost immediately after inducer readdition, the STXA2 strain initiated the recovery process by mobilizing reserve polymers and starting respiration. This allowed the restoration of protein synthesis, gene transcription, and reconstitution of cellular structures to reestablish cell growth. In summary, our study highlights the role of TrxA in anabolic metabolism by coordinating cyanobacterial physiology through translation regulation.

## Results

### Decrease of TrxA levels leads to the increased cell size and glycogen and cyanophycin accumulation

**Figure 1.**
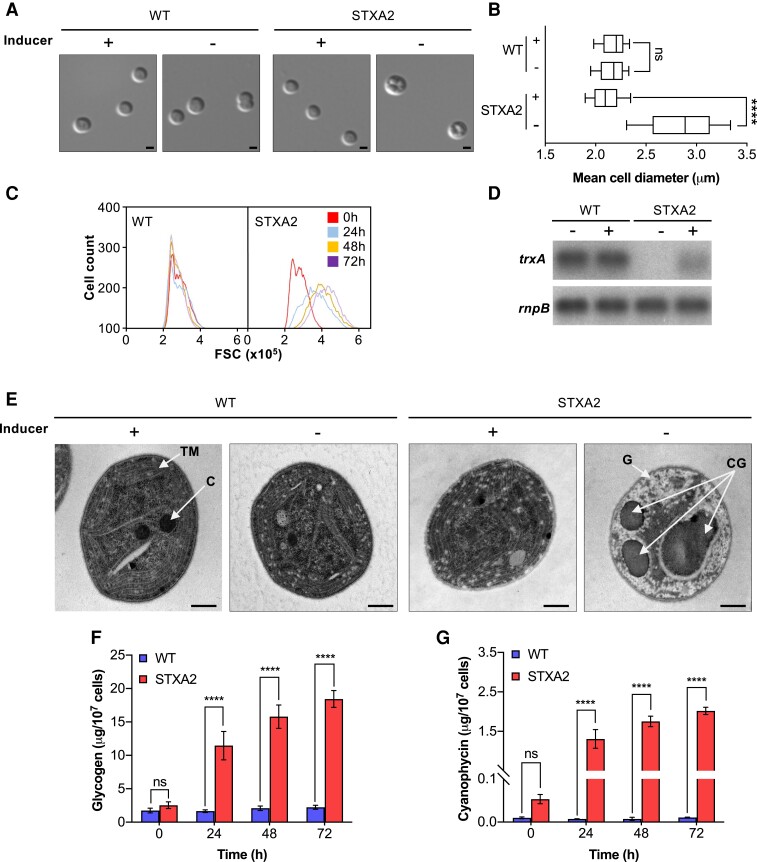
Morphological analysis and quantification of reserve polymers in WT and STXA2 cells. **A)** Light microscopy and **E)** transmission electron images of WT and STXA2 cells before (+0 h) and after inducer removal (−48 h). TM, thylacoid membrane; C, carboxysome; G, glycogen; CG, cyanophycin granules. **B)** Mean cell size from 200 measurements for each strain before (+) and 48 h after inducer removal (−). In box plots, the center line represents the median, the box limits denote the upper and lower quartiles, and the whiskers indicate the interquartile range. Data were obtained by light microscopy using ImageJ software. Scale bars in **A)** and **E)**: 0.5 and 1 *μ*m, respectively. **C)** Flow cytometric analysis of the cell size of WT and STXA2 cells before (0 h) and after inducer removal (24, 48, and 72 h). **D)** Northern blot analysis of *trx*A before (+) and 48 h after inducer removal (−). Total RNA was isolated from WT and STXA2, and 5 *µ*g were blotted and hybridized with *trx*A and *rpn*B probes (loading control). **F)** Glycogen and **(G)** cyanophycin content in the WT and STXA2 cells before (0 h) and after inducer removal (24, 48, and 72 h). The error bars in **B)**, **F)** and **G)** represent the SD of the mean values from 3 independent experiments. The asterisks indicate significant difference using 2-way ANOVA test; ****P < 0.000; ns, no significant difference.

As described above, STXA2 is a conditional mutant of TrxA that is unable to maintain the cellular redox state, affecting the function of essential processes such as photosynthesis, carbon fixation, and oxidative stress. Under conditions of TrxA depletion, the mutant disassembles the photosynthetic apparatus and arrests growth (Mallén-Ponce et al. 2021). We examined alterations in the morphology, cell size, and ultrastructure in WT and STXA2 cells to try to explain the physiological changes that STXA2 undergoes. A comparative analysis of WT and STXA2 cells using flow cytometry and light microscopy revealed similar results in the presence of inducer (Fig. 1, A to C). After 48 h of inducer removal and with undetectable *trx*A levels (Fig. 1D), a gradual increase in cell size was observed, with an ∼1.5-fold increase in apparent diameter relative to the initial time point (Fig. 1, A to C). Transmission electron microscopy analysis revealed that the ultrastructure of the thylakoid membrane (TM) in WT and STXA2 cells appeared similar in the presence of inducer (Fig. 1E). In both strains, chlorophyll-mediated and phycobilisome-mediated fluorescence was distributed at the cell periphery in the mid-plane (Supplementary Fig. S1). In contrast to STXA2 and after inducer removal, the internal TM system is almost completely lost, and remnants of the TMs remain as disordered sheets that traverse the cytoplasm (Fig. 1E). Additionally, carboxysomes (C) were almost absent, and large inclusion bodies were observed and possibly identified as cyanophycin granules (CGs) in STXA2 in this situation (Fig. 1E). This massive accumulation of CG occupied ∼30% to 60% of the cell surface (Fig. 1E; Supplementary Fig. S2). In addition to the accumulation of cyanophycin, the accumulation of large amounts of glycogen (G) was found in STXA2 cells.

Additionally, the probable accumulation of CG was also observed by light microscopy (Fig. 1A). We determined glycogen and cyanophycin levels over time and observed that, in the presence of inducer, STXA2 had ∼1.5-fold and 4-fold higher levels of glycogen and cyanophycin, respectively, compared with WT (Fig. 1, F and G; Supplementary Fig. S3). Upon inducer removal, there was a strong increase in glycogen and cyanophycin levels, especially during the first 24 h in STXA2. After 48 h, the amount of glycogen and cyanophycin per cell was 7-fold and 40-fold higher, respectively, compared with the initial time (Fig. 1, F and G; Supplementary Fig. S3). The accumulation of storage compounds and the increase in cell size are likely related to inhibition of cell growth, which was completely stopped after 48 h of inducer removal (Mallén-Ponce et al. 2021).

### TrxA is required for the control of protein synthesis

**Figure 2.**
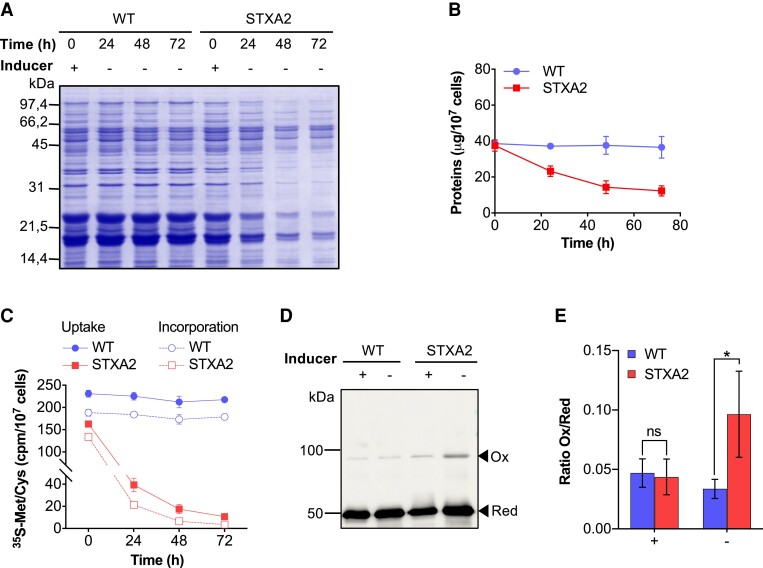
Effects of decreased TrxA levels on protein synthesis in the STXA2 strain. **A)** Coomassie Brilliant Blue–stained SDS-polyacrylamide gel of cell-free extracts from WT and STXA2 before (+0 h) and after inducer removal ([−] 24, 48, and 72 h). Samples equivalent to 4 × 10^7^ cells were taken at the indicated time and fractionated by reducing SDS-PAGE. **B)** Total protein content and **C)**^35^S-Met/Cys uptake and incorporation in WT and STXA2 cells before (+0 h) and after inducer removal (24, 48, and 72 h). Cpm, counts per minute. **D)***In vivo* redox state of EF-Tu. Western blot analysis was performed using cells from WT and STXA2 strains collected before (+) and 48 h after inducer removal (−). Cells were treated with NEM, cell extracts were obtained and proteins were fractionated by nonreducing SDS-PAGE. Ox, oxidized; Red, reduced forms. **E)** The ratio of oxidized to reduced EF-Tu fraction in the WT and STXA2 strains is shown. The error bars in **B)**, **C)**, and **E)** represent the SD of the mean values from 3 independent experiments. The asterisks indicate significant difference using 2-way ANOVA test; *P < 0.05; ns, no significant difference.

Accumulation of cyanophycin and glycogen has been reported to be triggered by different stress conditions or, more specifically, by inhibition of protein synthesis (Allen et al. 2005; Elbahloul et al. 2005). Therefore, the accumulation of these storage compounds in response to low TrxA levels in STXA2 could be associated with a substantial limitation in protein synthesis. To initially validate this hypothesis, the total protein content of the WT and STXA2 strains was compared at 0 h and after inducer removal (24, 48, and 72 h; Fig. 2, A and B). In the presence of the inducer, the protein content in STXA2 was slightly lower than in WT, while it was reduced to approximately half the initial level in STXA2 24 h after inducer removal. Subsequently, to directly analyze the effect of TrxA downregulation on protein synthesis, we then monitored methionine and cysteine (^35^S-Met/Cys) uptake and ^35^S-Met/Cys incorporation into proteins in WT and STXA2 strains over time. In the presence of inducer, both rates were 30% lower in STXA2 compared with WT, which could be related to a delay in cell growth (Fig. 2C). These differences between the 2 strains were maintained even after different incubation times with ^35^S-Met/Cys (Supplementary Fig. S4, A and B). Furthermore, a 4-fold and 10-fold and 6.5-fold and 20.5-fold decrease in ^35^S-Met/Cys uptake and incorporation was observed, respectively, 24 and 48 h after inducer removal (Fig. 2C). Both processes were substantially affected by the decrease in TrxA levels and appear to be closely related. To determine whether a limitation in protein synthesis leads to a reduction in ^35^S-Met/Cys uptake, we added the translation inhibitor lincomycin to both WT and STXA2 cells in the presence of inducer (Supplementary Fig. S4C). In both strains, the effect of lincomycin on blocking ^35^S-Met/Cys incorporation and uptake was drastic. In addition to the above, STXA2 cells showed a more pronounced decrease in ^35^S-Met/Cys incorporation into proteins than in ^35^S-Met/Cys uptake. The ratio of ^35^S-Met/Cys incorporation to uptake decreased from 0.82 in STXA2 in the presence of inducer to 0.37 after 48 h of inducer removal, whereas it remained constant in the WT strain (Supplementary Fig. S4D).

We also analyzed whether the total amino acid pool was altered by TrxA depletion. Indeed, inducer removal caused a change in the amino acid pool in STXA2 due to a limitation of protein synthesis, possibly triggering cyanophycin accumulation (Supplementary Fig. S5). In the presence of inducer (0 h), the levels of aspartate, serine, alanine, glycine, valine, proline, isoleucine, and leucine were ∼1.5-fold to 2.5-fold higher than those of WT, while phenylalanine and tryptophan levels were ∼1.5-fold to 2-fold lower than those of WT (Supplementary Fig. S5). After inducer removal (48 h), alanine, serine, and glycine levels increased up to 3-fold to 4-fold, while valine, leucine, threonine, glutamine, isoleucine, and asparagine levels increased 2-fold to 3-fold relative to the initial time.

Several proteomics studies have identified potential TrxA targets involved in different biological processes, including protein synthesis (Pérez-Pérez et al. 2006; Mata-Cabana et al. 2007). In *Synechocystis*, ROS have been shown to inactivate translational elongation, and EF-Tu has been identified as a target. Since oxidized EF-Tu can be reduced and reactivated by Trx *in vitro* and *in vivo* (Yutthanasirikul et al. 2016), we analyzed the redox state of EF-Tu in the STXA2 strain. We added *N*-ethylmaleimide (NEM) to cell suspensions before and after inducer removal and analyzed samples by Western blotting using specific antibodies. In the presence of inducer, only a small fraction of EF-Tu was found in the oxidized fraction/dimer fraction in both WT and STXA2. After 48 h of inducer removal, an increase in the oxidized fraction of EF-Tu was observed in the STXA2 strain (Fig. 2D), resulting in an increase in the oxidized/reduced ratio compared with the WT strain (Fig. 2E).

### Restoring TrxA levels enables a return to a normal cellular state

**Figure 3.**
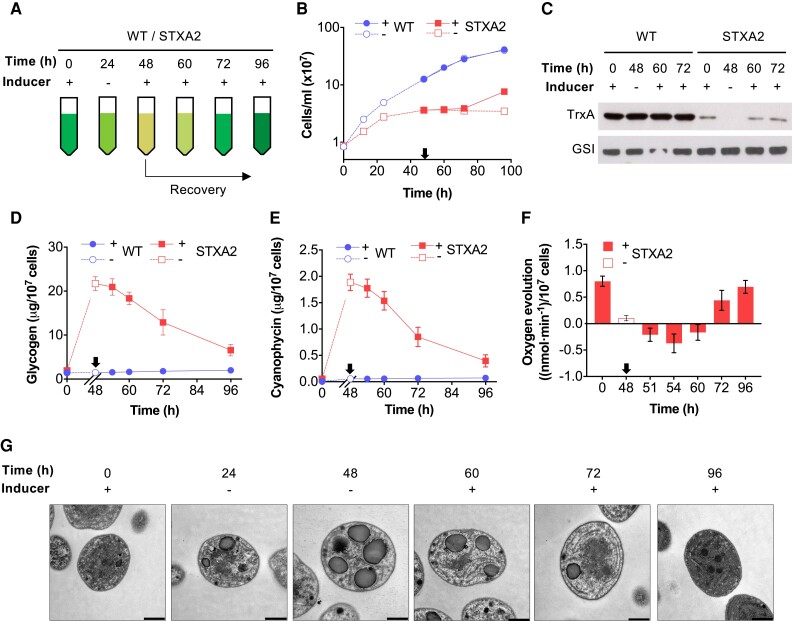
The analysis of cell growth, morphology, cyanophycin and glycogen levels and oxygen evolution during the recovery of STXA2. **A)** A schematic representation of the experimental procedure to analyze the effect of inducer readdition (recovery) to the STXA2 strain after 48 h without inducer. **B)** Growth curve of the STXA2 strain after inducer addition to cells from which inducer had been withdrawn for 48 h. The WT strain was used as a control. **C)** TrxA protein levels in WT and STXA2 after inducer readdition. Glutamine synthetase I (GSI) protein was used as a control. **D)** Glycogen and **E)** cyanophycin levels in WT and STXA2 after inducer readdition. **F)** Oxygen evolution in STXA2 cells after inducer readdition. **G)** Transmission electron microscopic images of STXA2 cells after inducer addition. The total time of 60, 72, and 96 h correspond to 12, 24, and 48 h after inducer readdition, respectively. Scale bar: 0.5 *μ*m. The error bars in **B)**, **D)**, **E)**, and **F)** represent the SD of the means values from 3 independent experiments. Inducer addition is indicated by arrows.

After 48 h of inducer removal, the cell color changed from blue-green to yellowish (Mallén-Ponce et al. 2021), resulting in arrested cell growth for up to 7 d in a dormant-like state. As glycogen and cyanophycin reserve polymers can be used as a source of carbon and nitrogen sources, we examined the effects of inducer readdition in STXA2 cells that had been starved for 48 h (Fig. 3A). The STXA2 strain showed recovery of cell growth after 24 h of induction (72 h in total; Fig. 3B). Additionally, the cell color of STXA2 cells changed from yellowish to blue-green cells within the first 24 h (Supplementary Fig. S6A). Accordingly, quantitative analysis of chlorophyll and phycobiliproteins showed a substantial increase after 24 h of induction (Supplementary Fig. S6, B and C). Interestingly, TrxA protein levels were restored after 12 h (60 h in total; Fig. 3C), although the number of cells did not increase at this time. Then, we analyzed the degradation of the reserve polymers during recovery. At 6 h after induction, there was a slight but clear decrease in glycogen and cyanophycin content, which continued until levels reached close to those of the WT (Fig. 3, D and E). Finally, to test whether both reserve polymers could be used as a carbon source, we analyzed oxygen evolution during recovery (Fig. 3F). In STXA2, traces of oxygen produced by residual photosynthesis were detected after 48 h of inducer removal. However, only 3 h after induction, STXA2 cells started to consume oxygen despite illumination. The mutant reached the maximum respiration rate after 6 h, and oxygen consumption was observed up to 12 h after induction. Furthermore, photosynthetic oxygen production was detected after 24 h of induction, consistent with the reappearance of photosynthetic pigments (Supplementary Fig. S6, B and C). Consequently, electron microscopic analysis showed a decrease in glycogen and cyanophycin during the recovery process (Fig. 3G). After 24 h of induction, STXA2 cells had reconstituted TMs in parallel stacks, and carboxysomes became visible.

**Figure 4.**
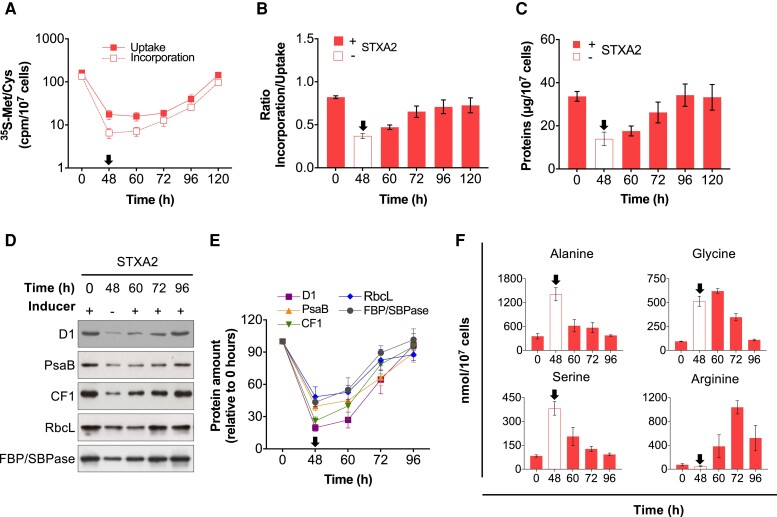
Effects of recovery of TrxA levels on protein synthesis in the STXA2 strain. **A)**^35^S-Met/Cys uptake and incorporation, **B)**^35^S-Met/Cys incorporation/uptake ratio, and **C)** total protein content in the STXA2 strain during the recovery. The time points of 60, 72, 96, and 120 h correspond to 12, 24, 48, and 72 h after inducer readdition, respectively. **D)** Western blot analysis of the indicated proteins. Samples equivalent to 4 × 10^7^ cells were taken in the presence of inducer (+0 h), 48 h (−) after inducer removal, and after inducer readdition (60, 72, and 96 h). Total proteins were isolated, resolved on SDS-PAGE, blotted, and incubated with specific antibodies. **E)** Quantification was performed on the indicated proteins during the recovery relative to the signal with the inducer (0 h). Quantifications were made using ImageJ software. **F)** Amino acid content in STXA2 cells in the presence of inducer (0 h), 48 h after inducer removal, and after inducer readdition (60, 72, and 96 h). The error bars in **A)**, **B)**, **C)**, **E)**, and **F)** represent the SD of the mean values from 3 independent experiments. Inducer addition is indicated by arrows.

Blocking protein synthesis leads to a strong accumulation of cyanophycin, which is a nitrogen-rich polymer of asparagine and arginine. We hypothesize that when TrxA levels are below 2% of WT, cyanophycin accumulates and can be used as an intracellular amino acid source to reactivate protein synthesis. To test this hypothesis, we analyzed the incorporation of ^35^S-Met/Cys into proteins and the uptake of ^35^S-Met/Cys into cells (Fig. 4A). Surprisingly, we observed low levels of both up to 72 h after induction in STXA2 cells, possibly related to the presence of cyanophycin and intracellular amino acids. In contrast to the previous results, the ratio of ^35^S-Met/Cys incorporation and ^35^S-Met/Cys uptake and total protein levels were fully recovered after 24 to 48 h of induction (72 to 120 h of total time; Fig. 4, B and C). Some of the photosynthetic and CB cycle proteins that had decreased after removal of the inducer returned to their initial levels during the recovery period (Fig. 4, D and E). Analysis of the levels of these proteins showed no significant changes in the abundance of the D1 subunit of PSII, the PsaB subunit of PSI, the RbcL subunit of the RuBisCo enzyme, and the FBP/SBPase enzyme during the first 12 h of recovery. However, an increase was observed between 24 and 48 h (72 to 96 h of total time) after inducer readdition (Fig. 4, D and E). After the addition of the inducer and subsequent restart of protein synthesis, there was a decrease in amino acid levels that had increased in the absence of the inducer (Fig. 4F; Supplementary Fig. S7), although recovery times varied. Arginine levels increased 20-fold after 24 h of induction due to cyanophycin degradation (Fig. 4F). Interestingly, aspartate levels increased only 1.7-fold after 24 h of induction (Supplementary Fig. S7). Finally, a change in the *in vivo* oxidation state of EF-Tu factor and 2-Cys Prx was observed after inducer readdition, resulting in a decrease in the oxidized/reduced ratio (Supplementary Figs. S8 and S9).

### The transcriptome of TrxA depletion

To gain further insight into the redox crisis caused by TrxA depletion, we analyzed the transcriptome of STXA2 cells with different levels of TrxA. Total RNA was extracted from WT and STXA2 cells in the presence of inducer and STXA2 cells after inducer removal (24 and 48 h) and inducer readdition (60 and 72 h). On the one hand, we compared the gene expression levels of the WT and STXA2 strains in the presence of inducer. On the other hand, we compared the gene expression response of STXA2 cells with inducer removal (24 and 48 h) and readdition (60 and 72 h). In this case, the fold changes in transcript levels were relative to the abundance of transcripts in STXA2 cells growing in the presence of inducer.

**Figure 5.**
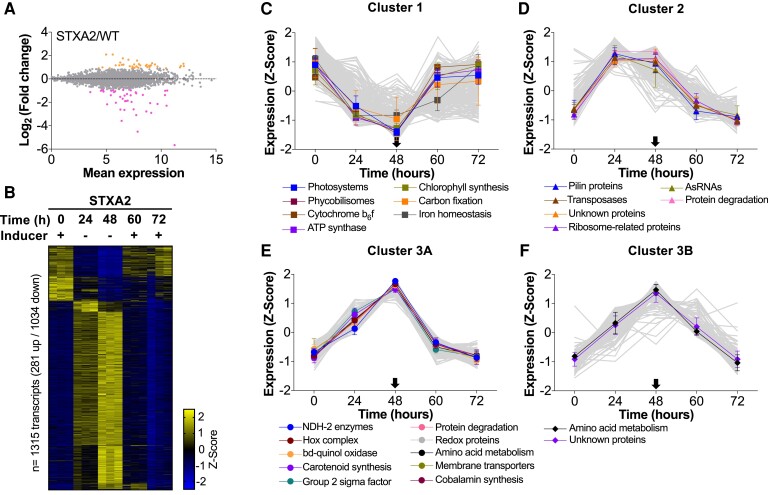
The transcriptomic analysis of the STXA2 strain after depletion and recovery of TrxA levels. **A)** MA plot of Log_2_-fold-change (STXA2/WT) versus mean expression in Log2 scale for each gene was generated using the DEseq2 package. Colored dots indicate differentially expressed genes with (|log_2_ FC ≥ 0.9 and adjusted *P*-value ≤ 0.05). **B)** Heat maps showing the *Z*-score for those transcripts that significantly changed in the STXA2 strain after inducer removal relative to the levels with inducer (0 h; |log_2_ FC| ≥ 0.9 and adjusted *P*-value ≤ 0.05). **C to F)** Representation of the major transcript groups in response to inducer removal and readdition. The error bars represent the SD of the mean values from 3 independent experiments. Inducer addition is indicated by arrows.

In the presence of the inducer, 302 transcripts showed a significant change between both strains (Fig. 5A; Supplementary Table S1). However, only 94 transcripts showed a significant change in their expression level [log_2_ fold change (FC)| ≥ 0.9 and adjusted *P*-value ≤ 0.05], with 37 genes upregulated and 41 genes downregulated in STXA2 compared with WT (Fig. 5A). All other transcripts were noncoding RNAs (ncRNAs) and antisense RNAs (asRNAs). Notably, most of the downregulated genes comprised the FurA regulon (Supplementary Table S2). On the other hand, transcripts encoding Rubisco and carboxysome operons, as well as metabolic (sll0944/CfrA) and transcriptional (sll1330/Rre37) regulators directly involved in carbon metabolism, were upregulated (Tabei et al. 2007; Muro-Pastor et al. 2020).

In the STXA2 strain, removal of the inducer and subsequent depletion of TrxA resulted in a robust transcriptional response. Statistical analysis identified 1,315 transcripts that showed a significant change in their expression levels (|log_2_ FC| ≥ 0.9 and adjusted *P*-value ≤ 0.05), with 281 upregulated genes and 1,034 downregulated genes (Fig. 5B; Supplementary Table S3). To provide a global view of the most relevant expression patterns, transcripts were grouped using the *k*-means clustering algorithm. The analysis of expression patterns and timing coincidence analysis allow the identification of differentially expressed genes associated with different functional categories, reflecting a chronological order in the transcriptome during inducer removal and readdition.

Genes that respond negatively are grouped into a large cluster, termed Cluster 1, and are related to photosynthesis, ATP synthesis, and CO_2_ fixation (Fig. 5C; Supplementary Table S4). Most of these genes are progressively repressed after inducer removal but recover their initial levels after 12 h of inducer readdition. Interestingly, genes encoding the D1 protein of PSII, whose protein levels decrease substantial, do not follow this pattern of gene expression. Several ncRNAs and asRNAs were also identified within this cluster. Among the ncRNAs that were downregulated after inducer removal, we found ncr0700/PmgR1, which regulates glycogen accumulation (de Porcellinis et al. 2016).

Genes that respond positively are grouped into various clusters according to the time course of the changes (Fig. 5, D to F; Clusters 2 and 3). Cluster 2 comprises many genes that reached peak expression after 24 h of inducer removal (Fig. 5D; Supplementary Table S5). The largest classes of Cluster 2 encode hypothetical or unknown proteins, which may be involved in similar cellular functions, as well as asRNAs. Other differentially expressed genes within this cluster encoded proteases, ribosome-related proteins, putative transposases, and pilin proteins. Cluster 3 contained genes whose expression levels increased progressively, reaching peak expression after 48 h of inducer removal. This cluster can be divided into 2 subgroups according to their time course during recovery: 3A (transcripts that return to initial levels after 12 h) and 3B (transcripts that return to initial levels after 24 h). Interestingly, Cluster 3A includes genes related to respiratory electron transport pathways (Type 2 NAD(P)H dehydrogenases and cytochrome bd-quinol oxidase), an alternative electron transport pathway (hydrogenase complex), and the carotenoid synthesis pathway (Fig. 5E; Supplementary Table S6). Other differentially expressed genes in this cluster include those involved in transcriptional regulation through Group 2 sigma factors, amino acid metabolism, and protein degradation. Genes encoding NblA1, NblA2, and NblC, which are adaptor proteins for the proteolysis of phycobilisome, were upregulated upon inducer removal. In contrast, Cluster 3B contained many transcripts encoding unknown proteins (Fig. 5F; Supplementary Table S7). Other transcripts within this cluster encode the SmpB protein, which is required for the rescue of stalled ribosomes (Karzai et al. 1999; Nonin-Lecomte et al. 2009), and 2 high-light–inducible polypeptides (HliA and HliC). Regarding asRNAs, most of them correspond with their sense mRNAs, with some exceptions, such as the upregulated asRNAs of pyruvate kinase (sll1275-as), aspartoacylase (slr1705-as), and ATP synthase protein I (sll1321-as). In all cases, asRNAs can play important regulatory roles, as previous work has shown (Dühring et al. 2006; Eisenhut et al. 2012; Sakurai et al. 2012).

## Discussion

Cyanobacteria have evolved robust redox regulation through Trxs, which profoundly influence metabolism in the daily light–dark cycle. TrxA is the only Trx present in all cyanobacteria (Mallén-Ponce et al. 2022) and plays an essential role in photosynthetic organisms, including *Synechocystis* (Muller and Buchanan 1989; Navarro and Florencio 1996; Chi et al. 2008; Wang et al. 2013). Our study aims to evaluate the critical role of TrxA in metabolism and cell growth using a conditional TrxA mutant. The results suggest that TrxA plays a key role in maintaining cell growth by influencing protein synthesis. A decrease in TrxA levels induces significant changes in phenotype, metabolism, and gene expression, resulting in a dormant-like state.

Interestingly, events during TrxA depletion in the STXA2 strain resemble the dormant state after nitrogen deprivation in cyanobacteria. In cyanobacteria, nutrient depletion leads to metabolic changes that result in decreased protein synthesis (Sauer et al. 2001), as well as the accumulation of reserve compounds such as cyanophycin during phosphorus starvation (Stevens et al. 1981; Watzer and Forchhammer 2018) and glycogen during nitrogen starvation (Collier and Grossman 1992). Under these conditions, cells enter a dormant chlorotic state as a survival strategy, and an analysis of transcriptome dynamics also revealed sophisticated regulation (Klotz et al. 2016). Similarly, TrxA depletion in the STXA2 strain led to a block in protein synthesis, redirecting carbon and nitrogen metabolism toward the synthesis of the reserve polymers glycogen and cyanophycin (Fig. 1). After more than 72 h in the absence of inducer and thus with low levels of TrxA, STXA2 cells were almost completely bleached, and components of the photosynthetic machinery were substantially reduced and entered a dormant-like state. Several studies have shown that cyanophycin is synthesized mainly in the presence of chloramphenicol, which is an inhibitor of protein synthesis. These studies have also shown that cyanophycin is produced through carbon and nitrogen assimilation, as well as degradation of cellular proteins (Allen et al. 1980, 2005). It is plausible that the degradation of phycobilisomes (PBS) and other protein complexes, after inducer removal, also contributes to cyanophycin and even glycogen formation.

Our results provide *in vivo* evidence that TrxA directly regulates protein synthesis (Fig. 2). Proteins comprise the largest mass fraction of all cellular components (Touloupakis et al. 2015) and are selectively produced in response to light (Singer and Doolittle 1975; Sarkar et al. 2019). Redox control of several proteins involved in translation, including the translation EF-G and EF-Tu, has been demonstrated in *Synechocystis* and *Synechococcus* (Ansong et al. 2014; Guo et al. 2014). Proteomic studies in *Synechocystis* and plant chloroplasts have suggested interactions between TrxA and EF-Tu/G (Balmer et al. 2003; Lindahl and Florencio 2003), and recent biochemical studies have also confirmed that both EFs can be reduced and activated by TrxA (Kojima et al. 2009; Yutthanasirikul et al. 2016; Toriu et al. 2023). There are 3 EF-G homologs in *Synechocystis*, and a mutation of one of them in which Cys-105 is replaced by a Ser residue improves protein synthesis under high light (Ejima et al. 2012). However, this improvement is limited to 20% possibly due to the presence of other EF-Gs. In contrast, there is only one copy of EF-Tu in the *Synechocystis* genome, which contains only one highly conserved cysteine residue (Cys-82) among bacteria (Nissen et al. 1999; De Laurentiis et al. 2011). Initial crystallographic analysis of *Escherichia coli* EF-Tu suggested that the cysteine residue is essential for nucleotide binding (Song et al. 1999). Furthermore, the substitution of cysteine for other residues resulted in an impaired ability of EF-Tu to bind both aminoacyl-tRNA and/or nucleotides, suggesting a role in stabilizing the ternary complex of EF-Tu, aminoacyl-tRNA, and GTP (Anborgh et al. 1992; De Laurentiis et al. 2011). In *Synechocystis*, EF-Tu is sensitive to oxidation when bound to GTP but hypersensitive when free (Yutthanasirikul et al. 2016). Interestingly, the addition of low levels of H_2_O_2_ only oxidized free EF-Tu by 30%, but it inactivates translational activity by more than 80%, suggesting that small changes in the redox state of EF-Tu strongly affect translational activity. Protein synthesis is inhibited under high light due to EF-Tu oxidation, and overexpression enhances protein synthesis under high-light conditions (Jimbo et al. 2019). In our study, we observed changes in the translational activity of the STXA2 strain in the presence of the inducer. Specifically, this activity was 30% lower than that of the WT strain (Fig. 2C). However, these changes were even more marked when the inducer was removed, resulting in a strong decrease in total protein content and translational activity in the STXA2 strain (Fig. 2, B and C). Analysis of the redox state of EF-Tu in STXA2 revealed a higher degree of EF-Tu oxidation in the absence of the inducer (Fig. 2, D and E), which explains the observed decrease in translational activity. Furthermore, when TrxA levels were partially restored by adding the inducer to STXA2 (Fig. 3C), recovery of protein content, translational activity (Fig. 4, A to C), and EF-Tu redox state (Supplementary Fig. S8) were observed, which explain the recovery of the WT-like phenotype (Fig. 3G; Supplementary Fig. S6). These results suggest that TrxA plays a critical role in the regulation of translational activity by modulating the redox state of EF-Tu. In addition, other translation-related proteins are likely to be oxidized in the STXA2 strain.

Similarly, to the physiological response, another notable and unexpected feature of the STXA2 strain is the transcriptomic response after inducer removal (Fig. 5). The dataset shows how TrxA levels affected the transcriptome at the genome-wide level. Compared with STXA2 in the presence of inducer, TrxA depletion resulted in an upregulation of transcripts related to respiratory or alternative electron transport, as well as those related to photoprotective functions. These included genes encoding high-light–inducible proteins (*hliA*, *hliB*, and *hliC*) and Nbl proteins that trigger PBS degradation. Interestingly, most of the highly upregulated transcripts belong to a hypothetical or unknown protein group, which together with asRNAs and ncRNAs could play an important role in the dormant state and its subsequent recovery. On the other hand, levels of photosynthesis-related transcripts decreased largely, except for *psbA* transcripts (Fig. 5C; Supplementary Table S3). Under these conditions, the limitation of protein synthesis in STXA2 causes a strong decrease in all photosynthetic proteins analyzed, including the D1 protein (Fig. 4, D and E). Our data are consistent with a recent study in which transcriptome and translatome analysis in *Synechocystis* revealed that photosynthesis is prioritized, with 34% ribosome occupancy, under normal growth conditions (Karlsen et al. 2022). The study also showed that most genes show similar transcriptomic and translatomic responses to reduced CO_2_ levels. Furthermore, transcriptome analysis during nutrient deprivation (Singh et al. 2003; Ludwig and Bryant 2012; Choi et al. 2016; Klotz et al. 2016) or dark acclimation (Saha et al. 2016) showed similar results to those presented in this study. All of these situations lead to a limitation of protein synthesis, but also of photosynthesis, which supplies TrxA with reducing equivalents, suggesting a link between TrxA and cell growth.

Proteomic analysis during light to dark transitions identified several redox-sensitive transcription factors in *Synechocystis* sp. PCC 6803 and *Synechococcus* sp. PCC 7002 (Ansong et al. 2014; Guo et al. 2014). Some of these factors, such as the response regulators A and B (RpaB and RpaA), Manganese sensing response regulator (ManR), Ferric uptake regulator A (FurA), or Photosynthetic Electron transport Dependent Regulator (PedR), a LuxR-type transcriptional regulator, are TrxA-interacting partners *in vitro* (Horiuchi et al. 2010; Kadowaki et al. 2015; Guío et al. 2021). RpaB is regulated in a thiol-redox state-dependent manner (Kato et al. 2022). Interestingly, analysis of the STXA2 transcriptome revealed changes related to regulon members of different transcription factors. FurA regulon, recently identified in *Synechocystis* (Riediger et al. 2021), showed a strong downregulation in the STXA2 strain even with inducer (Fig. 5C; Supplementary Table S2). Bacteria sense cytosolic Fe^2+^ in a concentration-dependent manner through its interaction with FurA. FurA binds iron, leading to a higher binding affinity for its target promoters, blocking their transcription. A recent study showed TrxA-dependent changes in the redox state of FurA in *Anabaena* sp. PCC7120 (Guío et al. 2021). Other transcription factors whose regulons have been established in *Synechocystis* are RpaB, Rubisco regulator (RbcR), and PedR (Nakamura and Hihara 2006; Riediger et al. 2019; Bolay et al. 2022). In the case of RbcR, changes in its target genes were observed in the induced STXA2 strain compared with the WT. In contrast, the target genes RpaB and PedR only showed changes in the STXA2 strain after inducer removal. Overall, these data suggest that TrxA could modulate these transcription factors *in vivo* depending on their levels and redox state. Future research will elucidate the *in vivo* role of TrxA in modulating transcription factor activity, and the STXA2 strain is an excellent tool.

**Figure 6.**
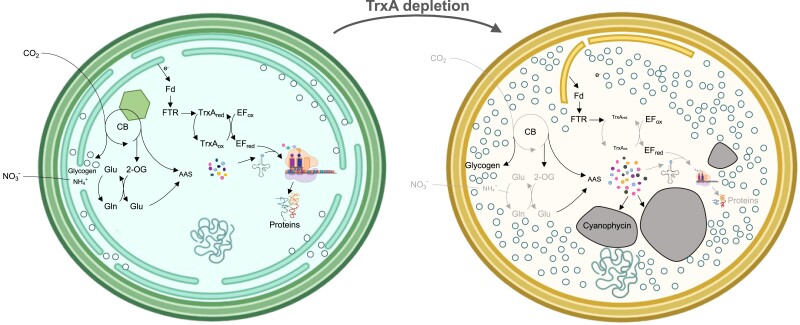
A schematic model of changes in STXA2 after TrxA depletion. STXA2 overview before (green left) and after (yellow right) TrxA depletion. In the absence of inducer, the STXA2 strain exhibits decreased TrxA levels, which affects the redox state of the EF-Tu factor. This results in a decrease in translational activity and a shift in carbon and nitrogen flux toward the storage of reserve polymers such as glycogen and cyanophycin. That is, TrxA depletion results in the arrest of anabolic metabolism, followed by the degradation of light-harvesting complexes, resulting in a dormant-like state. Ox, oxidized; Red, reduced forms; Glu, glutamate; Gln, glutamine; 2-OG, 2-oxoglutarate; Fd, ferredoxin; FTR, ferredoxin thioredoxin reductase.

The proposed model for the STXA2 strain under TrxA depletion conditions shows that TrxA has a strong effect on cellular processes. It leads to protein synthesis arrest, reduced photosynthesis, and accumulation of reserve polymers and amino acids (Fig. 6). These findings highlight the critical role of TrxA in the regulation of essential processes such as photosynthesis and carbon and nitrogen metabolism. However, further studies are needed to understand the metabolic consequences of TrxA depletion and to elucidate its functional relationships with other proteins and transcription factors.

## Materials and methods

### Growth conditions

*Synechocystis* sp. PCC 6803 cells were grown photoautotrophically at 30 °C in BG11 medium (Rippka 1988), supplemented with 1 g L^−1^ NaHCO_3_ (BG11C) and bubbled with 1% (v/v) CO_2_ in air, and under continuous illumination (50 *μ*mol photons m^−2^ s^−1^). For plate culture, 1% (w/v) Bacto-agar (Difco) and antibiotics (50 *µ*g mL^−1^ of nourseothricin and 2.5 *µ*g mL^−1^ of spectinomycin/streptomycin) and 1 mM arsenite (NaAsO_2_) were added when required. To perform experiments without arsenite (inducer), WT and STXA2, cells previously exponentially grown in media containing 1 mM arsenite, were harvested by centrifugation (4,300 *× g* for 5 min), washed twice, and then resuspended in BG11C medium without inducer.

### Glycogen content determination

Glycogen content was determined as described by Xu et al. (2013) with some modifications. A total of 10^8^ cells were harvested, washed twice with Milli-Q deionized water (Merck Millipore), and resuspended in 300 *µ*L of 30 mM sodium acetate pH 5.2. Cells were then disrupted using glass beads (0.15 to 0.25 mm) in a Mini-Beadbeater (BioSpec Products) by 2 vortexing cycles for 1 min followed by 5 min on ice. The extract was recovered and boiled for 20 min, 100 *µ*L was enzymatically hydrolized to glucose with 10 U amyloglucosidase from *Aspergillus niger* (Sigma-Aldrich) and 100 *µ*L was treated with Milli-Q deionized water as control. Finally, both aliquots were then incubated overnight at 55 °C. A commercial glycogen calibration curve was also prepared. Released glucose was determined in all aliquots using the glucose oxidase/peroxidase method (Sigma GAGO-20).

### Cyanophycin content determination

Cyanophycin content was determined according to Elbahloul et al. (2005) with some modifications. A total of 10^9^ cells were harvested, resuspended in 1 mL of 100% acetone, and incubated for 30 min at room temperature with gentle agitation. The samples were then centrifuged (15,000 *× g*, 4 °C for 15 min), and the acetone was carefully removed. To solubilize cyanophycin, the precipitates were resuspended in 1.5 mL of 0.1N HCl and incubated at 60 °C for 60 min with gentle agitation. The samples were centrifuged (15,000 × *g*, 4 °C for 15 min) to remove cellular debris, and the supernatants were transferred to a new tube. After the addition of 720 *µ*L of 0.1 M Tris/HCl pH 8.0, the samples were incubated at 4 °C for 40 min and centrifuged (15,000 × *g*, 4 °C for 15 min) to precipitate cyanophycin. Finally, the supernatants were removed, and the precipitates were resuspended in 500 *µ*L of 0.1N HCl. The cyanophycin content was then estimated by arginine determination according to the Sakaguchi reaction modified by Messineo (1966).

### Protein content determination and Western blot analysis

A total of 10^9^ cells were harvested, and crude extracts were obtained using glass beads. Total cellular protein was determined by the Lowry method (Markwell et al. 1978). For Western blot analysis, proteins were fractionated in 10% to 15% (w/v) sodium dodecyl sulfate polyacrylamide gel electrophoresis (SDS-PAGE) and transferred to nitrocellulose membranes (Bio-Rad). Blots were blocked in phosphate-buffered saline containing 0.1% (v/v) Tween 20% and 5% (w/v) nonfat dry milk and incubated with the required antibody. Antisera from the laboratory collection were used at the dilutions described in Lindahl and Florencio (2003) and Mallén-Ponce et al. (2021), and anti–EF-Tu from Nishiyama’s laboratory was used at 1:5,000. The ECL Prime Western Blot Detection Reagent (GE Healthcare) was used to detect different antigens with secondary antirabbit antibodies (1:25,000; Sigma-Aldrich).

### Microscopy

For light microscopy, the samples were observed in a Leica DM6000B fluorescence microscope and photographed with an ORCA-ER camera (Hamamatsu). Analysis was performed using the Leica Application Suite Advanced Fluorescence. For fluorescence microscopy, samples were observed using a laser-scanning confocal microscope (Leica TCS SP5). Cellular chlorophyll fluorescence was imaged by excitation with the 488-nm laser line of an argon laser using a 670- to 720-nm emission range. Images were treated with the Leica Application Suite Advanced. For the line profiles, intensities were extracted by image thresholding of the chlorophyll auto-fluorescence channel in Image J software. For electron microscopy, cells were prepared as described in Muro-Pastor et al. (2020). All samples were analyzed on a Libra 120 transmission electron microscope (Zeiss), and digital images were acquired with an on-axis mounted TRS camera.

### FACS analysis

Flow cytometry was performed on a BD Influx Fluorescence Activated Cell Sorter (FACS, BD Biosciences). Data were plotted on a 2D graph (*x*-axis, forward scatter [FSC] or side scatter [SSC]; *y*-axis, number of cells). FSC and SSC were plotted on a linear scale, and data were analyzed using FlowJo software (FlowJo LLC).

### Radioactive labeling of proteins in vivo

One milliliter of cells was placed in 10-mL glass tubes, and ^35^S-Met/Cys mixture (American Radiolabeled Chemicals) was added to a final concentration of 10 *μ*Ci. Samples were then placed under the same growth conditions. Two 500-*μ*L aliquots were taken at specified times, one for the analysis of ^35^S-Met/Cys uptake into cells and the other for ^35^S-Met/Cys incorporation into proteins.

For the analysis of ^35^S-Met/Cys uptake in cells, 500 *μ*L of BG11C containing nonradioactive Met/Cys at 1 mg mL^−1^ was added to the specific sample and was immediately placed on ice to end labeling. The medium was removed by vacuum filtration using a glass microfiber filter and washed 3 times with 5 mL of BG11C. Finally, the filters were immersed in scintillation fluid and counted on an LS 6000 IC scintillation counter (Beckman Instruments). For the analysis of ^35^S-Met/Cys incorporation into proteins, 500 *μ*L of a mixture of 20% TCA (w/v) and 2% SDS (v/v) was added to the specific sample and was immediately placed on ice for 1 h. The medium was removed by vacuum filtration using a glass microfiber filter and washed twice with 4 mL of 10% TCA (w/v) and once with 4 mL of ice-cold acetone. Finally, the filters were immersed in scintillation fluid and counted in the above-mentioned scintillation counter. Lincomycin (250 *μ*g mL^−1^) was used as a control to arrest protein synthesis.

### Amino acid pools quantification

Amino acid pools were quantified by reversed phase HPLC (Heinrikson and Meredith 1984). A total of 10^9^ cells were harvested, lyophilized, and resuspended in 400 *μ*L of 0.1 M HCl. After incubation on ice for 60 min, samples were centrifuged (15,000 × *g*, 4 °C and 15 min) to remove cell debris. Finally, 60-*μ*L samples were derivatized using the PICO TAG system (ethanol:H_2_O:triethylamine:phenyl isothiocyanate,7:1:1:1), dried, and resuspended in diluent (2 M Na_2_PO_4_, 10% [v/v] H_3_PO_4_, and 5% [v/v] acetonitrile). Separation was performed on a LiChrospher 100 RP-18 column (Merck KGaA). Amino acids were detected by absorbance and quantified using an amino acid standard mixture (Sigma-Aldrich).

### Redox state of EF-Tu in vivo

Alkylation assays were performed using NEM as described in Yutthanasirikul et al. (2016). Briefly, 10-mM NEM was added to 20 mL of cells grown at OD_750 nm_ 0.5, before and after inducer removal, and incubated for 20 min. Cells were harvested by centrifugation and disrupted by homogenization with glass beads in Buffer A (50 mM Tris-HCl, pH 8.0, 50 mM NaCl, and 10 mM of NEM), and cell extracts were obtained. Protein in cell extracts was precipitated with 10% TCA (w/v) for 1 h on ice and washed twice with ice-cold acetone. Proteins were then resuspended in Buffer B (50 mM Tris-HCl pH 8.0, 50 mM NaCl 2% [w/v], and SDS 7.5% [v/v]). For Western blot, proteins were resolved by 10% nonreducing SDS-PAGE and transferred to a nitrocellulose membrane, and EF-Tu was immunologically detected with an antiserum against EF-Tu.

### RNA extraction and transcriptomic analysis

Total RNA extraction was performed as described in García-Domínguez and Florencio (1997). Subsequently, the quantity and quality of total RNA were then assessed using RNA electropherograms acquired on an Agilent 2100 Bioanalyzer, and library construction of cDNA molecules was performed using Illumina Kapa Stranded Total RNA with a Ribo-Zero Library Preparation Kit. The resulting DNA fragments (DNA library) were sequenced on an Illumina HiSeq 4000 platform using 150-bp paired-end sequencing reads. Reads were aligned against the NCBI genome sequence for *Synechocystis* (NC_000911.1, NC_005229.1, NC_005230.1, NC_005231.1, and NC_005232.1) using Bowtie2 (Langmead and Salzberg 2012). Transcript assembly and estimate of gene expression were performed using StringTie (Pertea et al. 2016). For comparison between WT and STXA2 strains in the presence of inducer, differential expression analysis was performed using DESeq2 package (Love et al. 2014), while differential expression analysis in the STXA2 strain after inducer removal and readdition was carried out using ANOVA package with Benjamini and Hochberg correction (Benjamini and Hochberg 1995) RNA-seq dataset is available at the Gene Expression Omnibus accession (GSE243518).

### Statistical analysis

The statistical method used, the number of experimental repeats, and the results of statistical analyses are described in the figure legends. Two-way ANOVA was used for multiple comparisons within a dataset, with significance set to a *P-*value <0.05. ANOVA analysis was performed with GraphPad Prism software. Significant differences were indicated by *P < 0.05, **P < 0.005, ***P < 0.00, and ns, with no significant difference.

### Accession numbers

Sequence data from this article can be found in the GenBank/EMBL data libraries under accession numbers: BAA10623.1, TrxA (slr0623); BAA18321.1, EF-Tu (sll1099); BAA10136.1, 2-Cys Prx (sll0755); BAA10018.2, CfrA (sll0944); BAA18647.1, Rre37 (sll1330); BAA17954.1, NblA2 (ssl0453); BAA17404.1, NblC (sll1968); BAA18450.1, SmpB (slr1639); BAA17209.1, HliA (ssl2542); and BAA17603.1, HliC (ssl1633).

## Supplementary Material

kiae101_Supplementary_Data

## Supplementary data

The following materials are available in the online version of this article.

**Supplementary Figure S1.** Chlorophyll auto-fluorescence intensity profiles of WT and STXA2 cells.

**Supplementary Figure S2.** Transmission electron micrographs of STXA2 cells.

**Supplementary Figure S3.** Cyanophycin levels in WT and STXA2 cells.

**Supplementary Figure S4.** 3^5^S-Met/Cys uptake and incorporation in WT and STXA2 cells.

**Supplementary Figure S5.** Effects of decreased TrxA levels on the amino acid content in the STXA2 strain.

**Supplementary Figure S6.** Analysis of chlorophyll and phycobiliprotein levels during STXA2 recovery.

**Supplementary Figure S7.** Effects of recovery of TrxA levels on the amino acid content in the STXA2 strain.

**Supplementary Figure S8.** Analysis of the in vivo redox state of EF-Tu during STXA2 recovery.

**Supplementary Figure S9.** Analysis of the in vivo redox state of 2-Cys Prx during STXA2 recovery.

**Supplementary Table S1.** Transcripts showed significant changes between WT and STXA2 strains.

**Supplementary Table S2.** Fur regulon.

**Supplementary Table S3.** Genes grouped into Cluster 1.

**Supplementary Table S4.** Genes grouped into Cluster 2.

**Supplementary Table S5.** Genes grouped into Cluster 3A.

**Supplementary Table S6.** Genes grouped into Cluster 3B.

**Supplementary Table S7.** STXA2 time course raw data.

## Dive Curated Terms

The following phenotypic, genotypic, and functional terms are of significance to the work described in this paper:

ATP CHEBI: CHEBI:15422

amino CHEBI: CHEBI:46882

protein CHEBI: CHEBI:36080

GTP CHEBI: CHEBI:15996

cluster CHEBI: CHEBI:33731

glycogen CHEBI: CHEBI:28087
