# Supplementary material for: Thioredoxin A regulates protein synthesis to maintain carbon and nitrogen partitioning in cyanobacteria
Source: Plant Physiol. 2024 Feb 22;195(4):2921–36. doi: 10.1093/plphys/kiae101 (PMC11288746; doi:10.1093/plphys/kiae101)
Supplement: kiae101_Supplementary_Data [file kiae101_supplementary_data.zip › Supplementary Figures S1-S9.pdf]

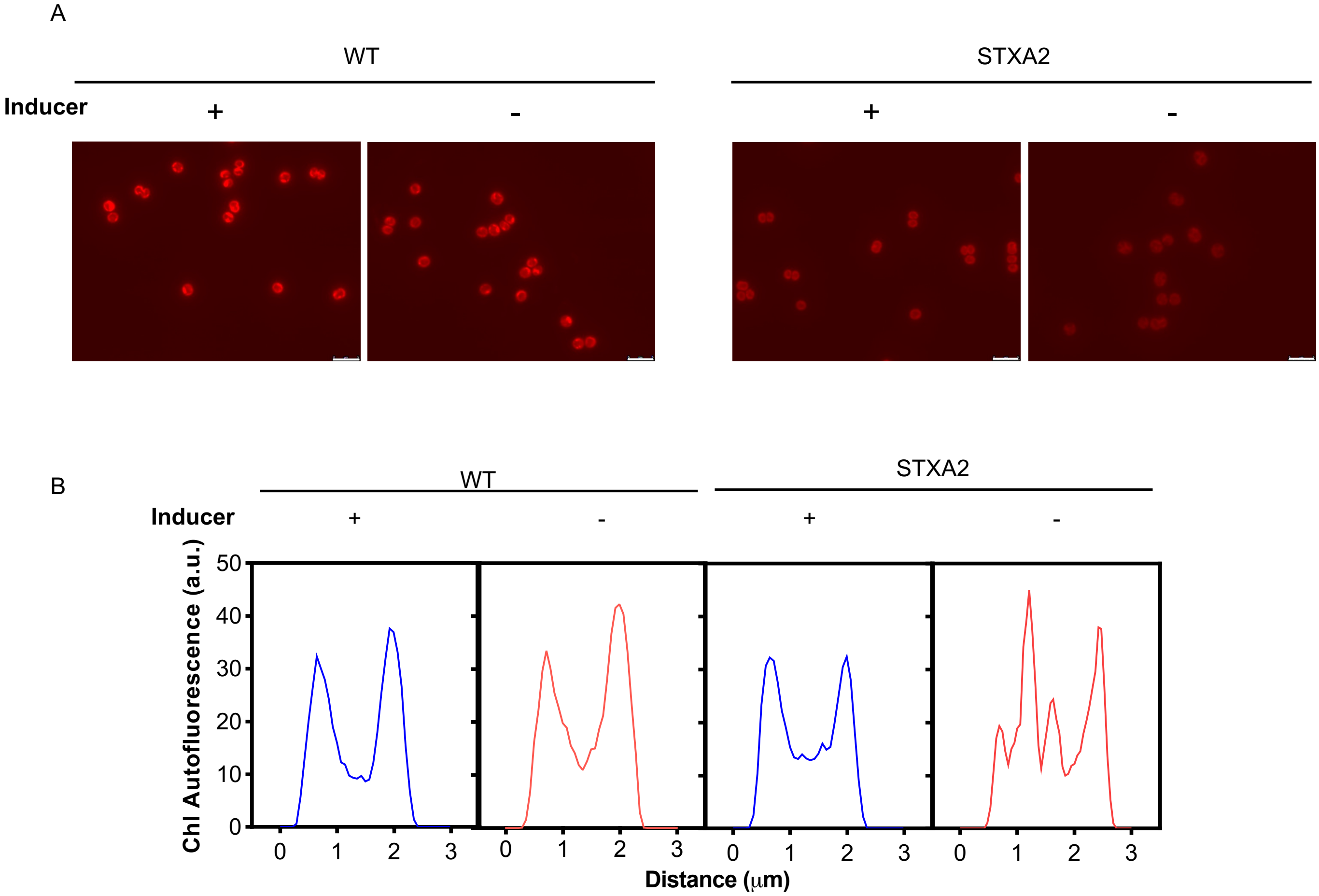


**Supplemental Figure S1. Chlorophyll autofluorescence intensity profiles of WT and STXA2 cells. (A)** Fluorescence live-cell imaging of wild type (WT) and STXA2 cells before (0h) and after inducer removal (48h). (B) Chlorophyll (Chl) fluorescence intensity profiles of WT and STXA2 cells. The white lines on the micrographs on the right of this figure show the scale bar: 5 μm.

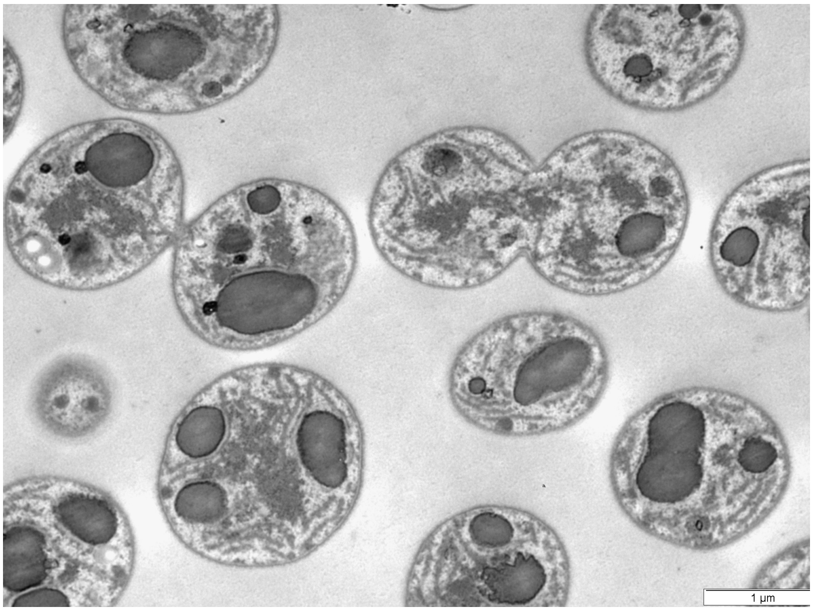


**Supplemental Figure S2. Transmission electron micrographs of STXA2 cells.** Transmission electron microscopy images of STXA2 cells after 48h of inducer removal. Scale bar in 1 µm.

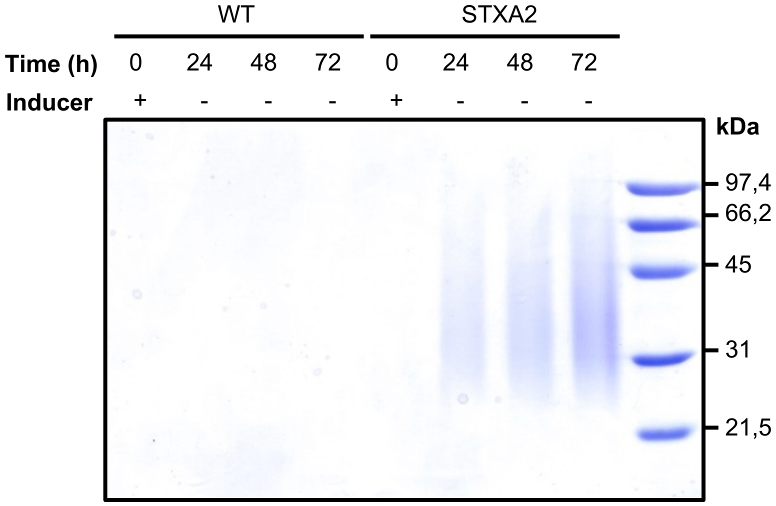


**Supplemental Figure S3. Cyanophycin levels in WT and STXA2 cells.** 15 µl of cyanophycin were isolated from $4.10^7$ cells of wild type (WT) and STXA2 cells before (+, 0h) and after inducer removal (24, 48 and 72h) and loaded onto a 12% SDS-PAGE and stained with CBB (Coomassie Brilliant Blue).

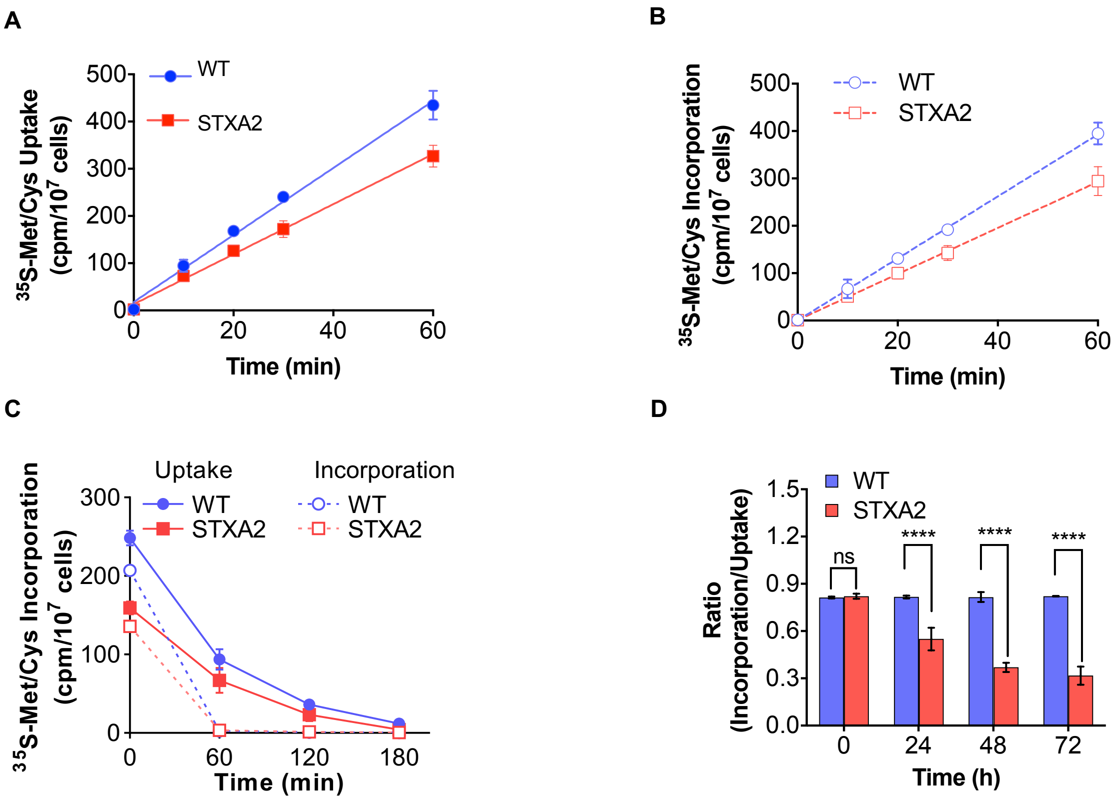


**Supplemental Figure S4. $^{35}$S-Met/Cys uptake and incorporation in WT and STXA2 cells.** (A) $^{35}$S-Met/Cys uptake and (B) $^{35}$S-Met/Cys incorporation over time in wild type (WT) and STXA2 cells with inducer. (C) $^{35}$S-Met/Cys uptake and incorporation in the WT and STXA2 cells with inducer after lincomycin addition. (D) Ratio $^{35}$S-Met/Cys incorporation/uptake in WT and STXA2 cells before (0h) and after inducer removal (24, 48 and 72h). Error bars represent the SD of the mean values from three independent experiments. Asterisks indicate significant differences using two-way ANOVA test ****$P<0.0001$, ns, no significant difference.

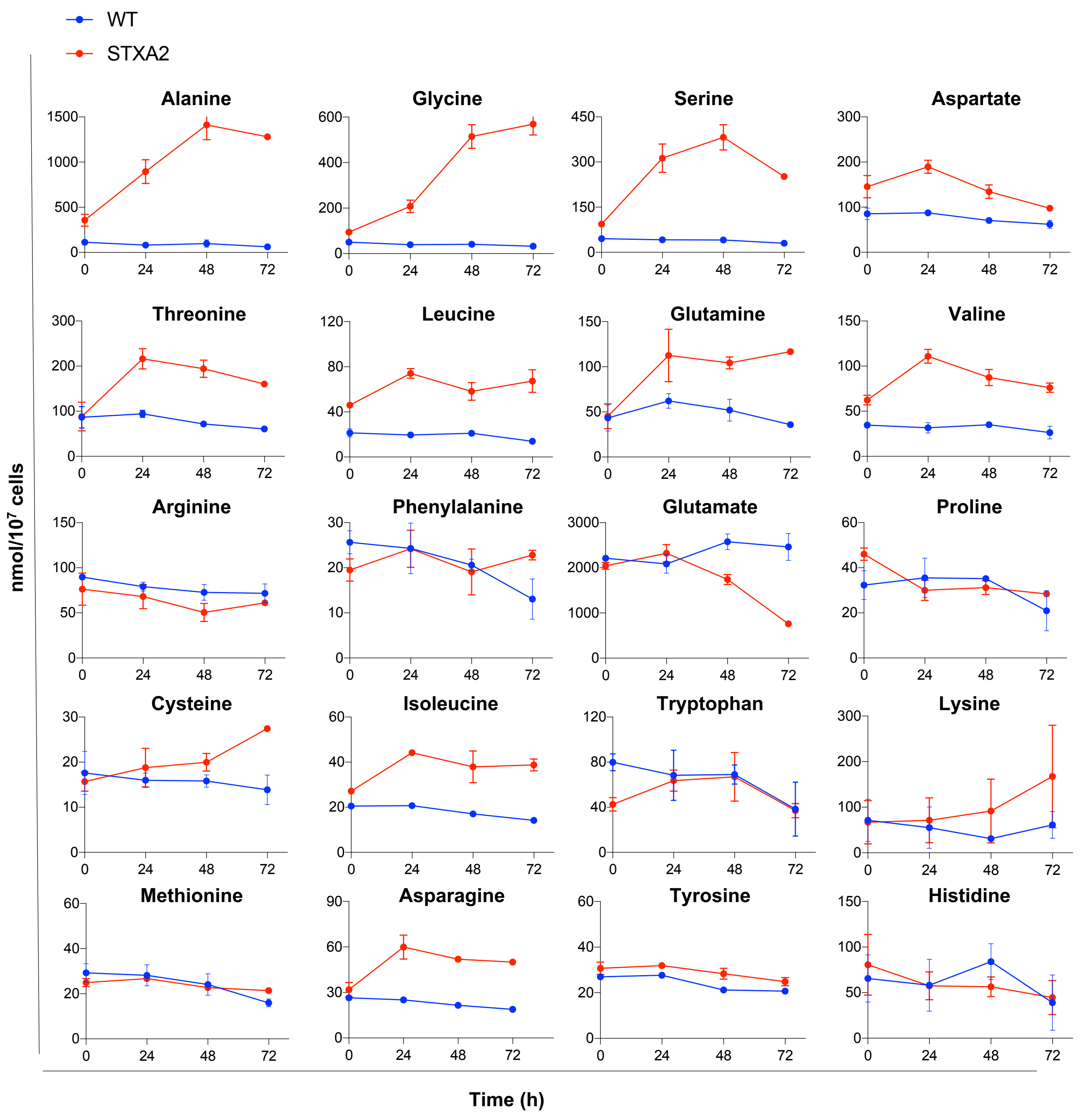


**Supplemental Figure S5. Effects of decreased TrxA levels on the amino acid content in the STXA2 strain.** Amino acid levels in wild type (WT) and STXA2 cells before (0h) and after inducer removal (24, 48 and 72h). . Error bars represent the SD of the mean values from three independent experiments.

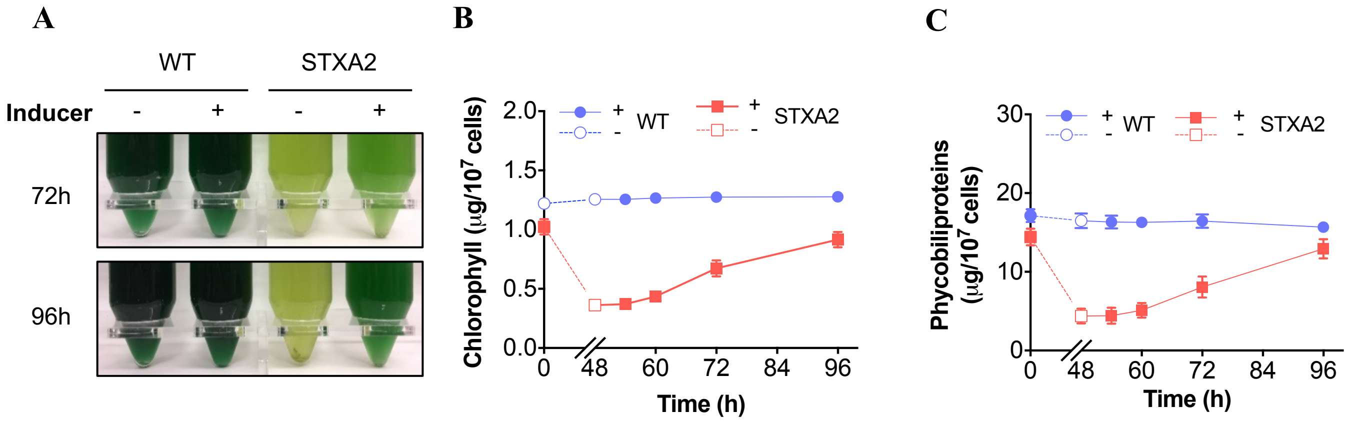


**Supplemental Figure S6. Analysis of chlorophyll and phycobiliprotein levels during STXA2 recovery.** (A) Photographic images of wild type (WT) and STXA2 strains after inducer re-addition. Time points of 72 and 96 h correspond to the 24 and 48 h after after inducer re-addition, respectively. (B) Chlorophyll and (C) phycobiliproteins contents in WT and STXA2 after inducer re-addition. Error bars represent the SD of the mean values from three independent experiments.

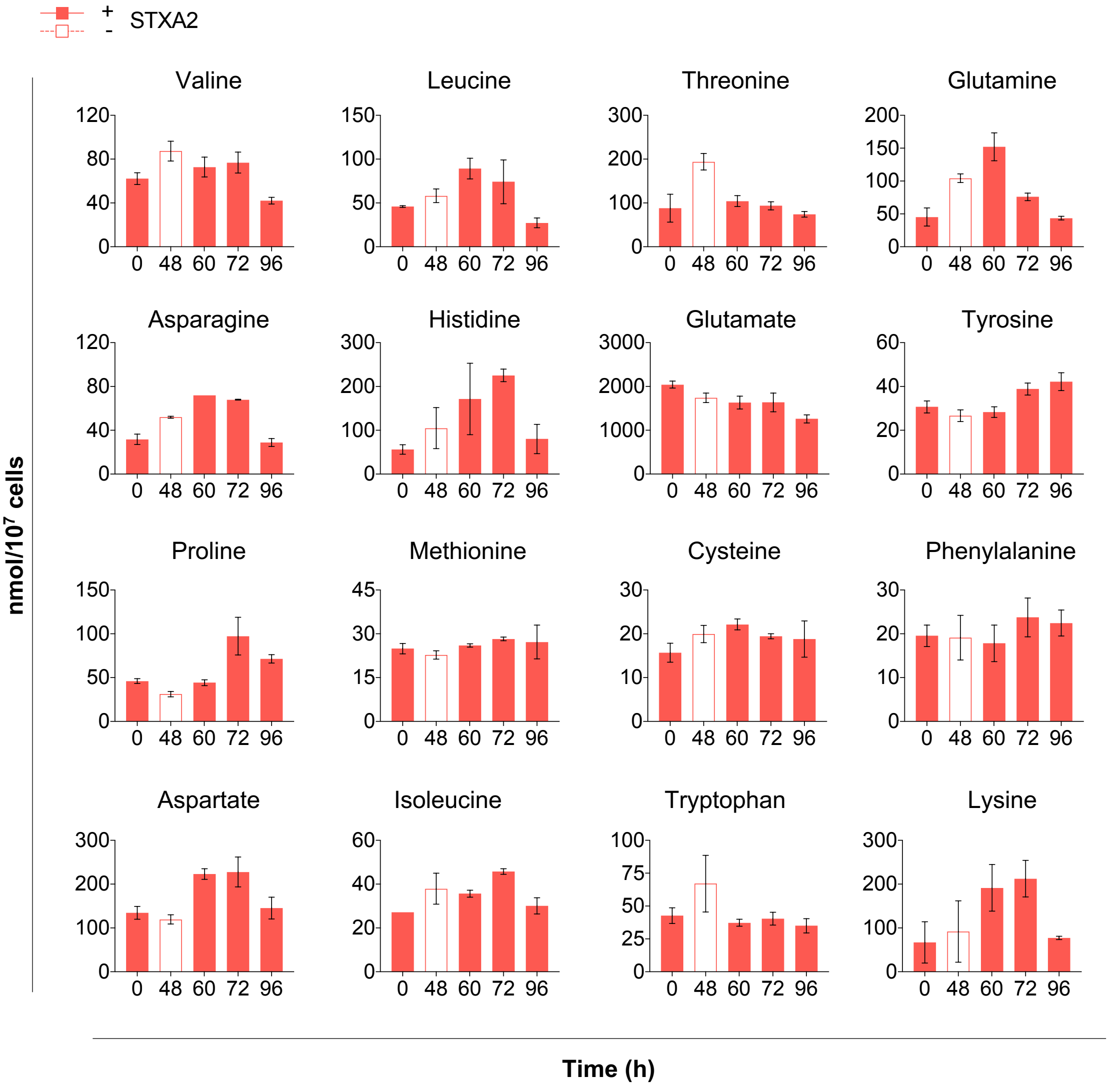


**Supplemental Figure S7. Effects of recovery of TrxA levels on the amino acid content in the STXA2 strain .** Amino acid content in STXA2 cells in presence of inducer (0h), 48 hours after inducer removal and after inducer re-addition (60, 72 and 96h). Error bars represent the SD of the mean values from three independent experiments.

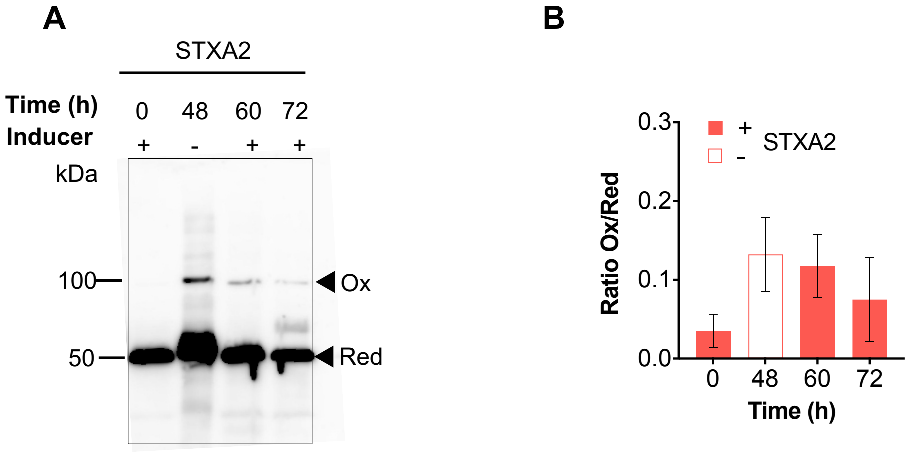


**Supplemental Figure S8. Analysis of EF-Tu of the *in vivo* redox state during STXA2 recovery.** (A) Western blot analysis was performed using cells of wild type (WT) and STXA2 collected before (0,48 h) and after inducer re-addition and then treated with N-ethylmaleimide (NEM). Cells extracts were obtained and separated by non-reducing SDS-PAGE and immunologically detected EF-Tu. Ox, oxidized and red, reduced forms. (B) The ratio of the oxidized to reduced EF-Tu fraction in the WT and STXA2 strains is shown. Error bars represent the SD of the mean values from three independent experiments.

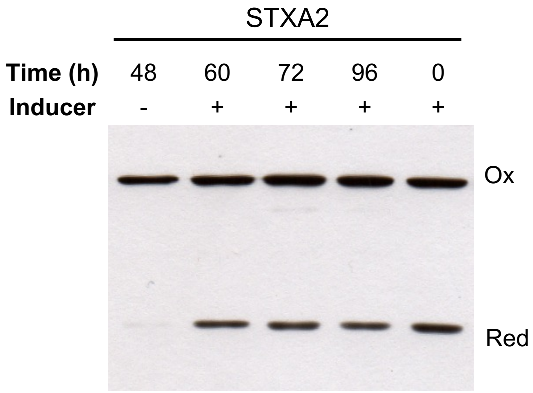


**Supplemental Figure S9. Analysis of the *in vivo* redox state of 2-Cys Prx during STXA2 recovery.** Cells were collected before (48 h) and after inducer re-addition (60, 72 and 96 h). *In vivo* redox state of 2-Cys Prx from exponentially growing STXA2 cells was used as a control (0). Ox, oxidized and red, reduced forms.
